# Effects of a Dietary Supplement with Barley Sprout Extract on Blood Cholesterol Metabolism

**DOI:** 10.1155/2015/473056

**Published:** 2015-05-25

**Authors:** A Ri Byun, Hyejin Chun, Jin Lee, Sang Wha Lee, Hong Soo Lee, Kyung Won Shim

**Affiliations:** ^1^Department of Family Medicine, Ewha Woman's University Mokdong Hospital, Ewha Woman's University School of Medicine, Seoul, Republic of Korea; ^2^Health Promotion Center, Ewha Woman's University Mokdong Hospital, Seoul 158-710, Republic of Korea

## Abstract

*Objective*. Barley sprout (*Hordeum vulgare* L.) contains 4.97% fat, 52.6% polysaccharide, and 34.1% protein along with a variety of vitamins, minerals, and polyphenolic compounds. Hexacosanol is one such compound from the barley leaf that might improve cholesterol metabolism by decreasing cholesterol synthesis. *Method*. Therefore, this study was conducted to investigate the effects of barley sprout extract on serum lipid metabolism in healthy volunteers (*n* = 51). Subjects were randomly divided into two groups: one group consumed a single capsule of barley leaf extract daily (*n* = 25, 42.48 ± 13.58 years) and the other consumed placebo capsules (*n* = 26, 40.54 ± 11.1 years) for 12 weeks. *Results*. After 12 weeks, total cholesterol and low-density lipoprotein- (LDL-) cholesterol were not lower in the barley sprout extract group compared to the placebo group (*p* = 0.415 and *p* = 0.351, resp.) and no differences in clinical or laboratory findings were observed between both groups. *Conclusion*. Our study failed to show significant lipid-lowering effects of barley sprout extract, possibly due to dosage, duration of therapy, and small sample size. Despite our nonsignificant findings, barley sprout has a possibility as a functional health food; therefore future research is needed.

## 1. Introduction

Increases in obesity, hypertension, diabetes, and hyperlipidemia today are due in part to increased consumption of a westernized diet that contains high amounts of calories and fat, lack of exercise, and an increase in environmental pollution [1]. If these chronic diseases are not managed properly, ultimately they may lead to atherosclerosis and an increase in the prevalence and mortality of cardiocerebrovascular disease. Accordingly, there has been a growing interest in reducing the prevalence and mortality of such chronic diseases [2]. When low density lipoprotein- (LDL-) cholesterol is lowered by 12% and total cholesterol by 8%, the risk of cardiocerebrovascular disease decreases by 19%; therefore, it may be crucial to manage hyperlipidemia with an appropriate lipid-lowering agent, such as a statin, which inhibits HMG CoA reductase, and lifestyle modifications [1]. However, from a health science perspective, people free from chronic disease who have blood cholesterol levels in the upper end of the normal range may progress into hyperlipidemia, leading to atherosclerotic complications afterward. Subsequently, those with upper-normal or high values of cholesterol may benefit from a supplement that improves blood lipid metabolism to prevent atherosclerotic complications.

A major food crop of humans [3] since its cultivation around 17,000–18,000 B.C., barley (*Hordeum vulgare*) has been the second most common crop (after rice) used in Korea, where people rely heavily on grains as a food source. Recent reports indicate that components of barley exhibit various positive physiological functions in the human body; thus, there has been growing attention to and research on barley [4]. Barley contains diverse physiologically active substances and the barley leaf contains lutonarin (isoorientin-7-*O*-glucoside) [5] and saponarin (flavone-C-glycosides) [6], which are known to have powerful antioxidant effects, as well as a high concentration of hexacosanol. A policosanol is a lipid alcoholic component composed of *β*-glucan, a water-soluble dietary fiber, and carbon numbers of 20–30, and is known to reduce cholesterol levels [7–10]. Research into the various physiological functions of barley and barley leaf extract reveals improved cholesterol and blood glucose levels [8, 11–13] along with antioxidant effects [5, 9, 10, 14]. In particular, the barley sprout, on which about 1–3 young leaves grow from a barley seed, contains high concentrations of diverse physiologically active substances to protect itself from external attacks and to germinate. Accordingly, the barley sprout has drawn more attention than barley seed or stalk [15]. Basic research that analyzed extracts obtained from a barley sprout confirmed the presence of various policosanol components; in particular, hexacosanol was present at the highest levels, comprising 62~80% of the extract [9]. When barley sprout extract is consumed in the form of a supplement, a high amount of hexacosanol is ingested; thus, barley sprout extract is expected to have a positive effect on blood cholesterol metabolism. However, no clinical trial in Korea has shown improved blood lipid metabolism as a result of taking barley sprout extract. Therefore, this study aimed to investigate the effects of barley sprout extract on blood lipid metabolism.

## 2. Methods

### 2.1. Subjects

**Figure 1:**
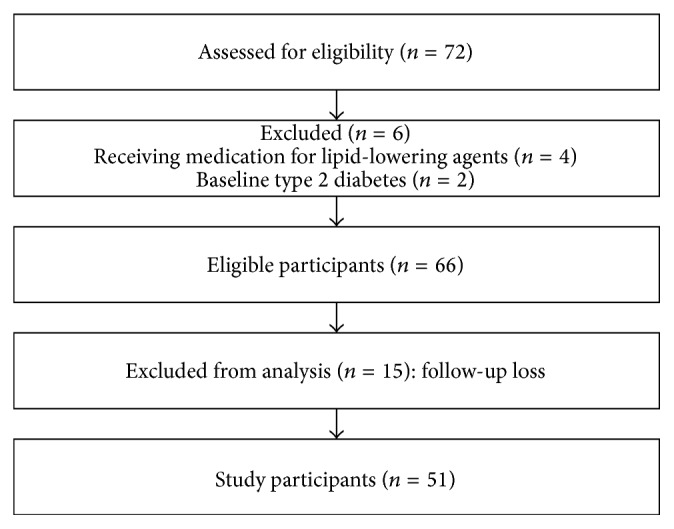
Flow sheet of the subjects selection.

Male and female adult applicants aged 20 to 65 were recruited through advertising at a university hospital. A total of 72 men and women aged between 20 and 65 were recruited through advertising at one university hospital from April to December 2013. Among these applicants, hypertensive and diabetic patients (*n* = 2) and patients taking a lipid-lowering agent, such as a statin or fenofibrate, for previously diagnosed hyperlipidemia (*n* = 4) were excluded from the trial through a preliminary questionnaire. In addition, those who took diet pills, diuretics, antidepressants, birth control pills, steroids, and/or female hormone drugs, all of which may affect weight or blood lipid metabolism; had hypersensitivity to food ingredients for the experiment; were pregnant or nursing; or had participated in other clinical trials within the last 4 weeks were excluded. Candidates with a blood pressure exceeding 140/90 mmHg, fasting glucose above 126 mg/dL, aspartate aminotransferase (AST) or alanine aminotransferase (ALT) elevated over 2.5 times the upper limit of the normal range, or serum creatinine levels above the normal range did not fit the standard for normal adults free of chronic disease and were also excluded. A total of 66 men and women qualified as the final research subjects for this clinical trial and the overall flow chart of this clinical trial is shown in Figure 1. This clinical trial was approved by the Institutional Review Board (IRB, No: ECT-12-34-08) of medical research ethics at Ewha Woman's University Mokdong Hospital, and all subjects provided written consent to the study procedures.

### 2.2. Experimental Protocol

#### 2.2.1. Health Examination

A medical history questionnaire was provided to all participants and the medical history of all the registered research subjects was elicited by a single doctor. The current and past diagnosis and medication history for hypertension, diabetes, cancer, and cardiocerebrovascular disease were investigated. Hypertension was defined as systolic/diastolic blood pressure exceeding 140/90 mmHg or current treatment with antihypertensive medication. Diabetes was defined as 8 h fasting blood glucose exceeding 126 mg/dL, glycated hemoglobin level exceeding 6.5% and confirmed through a blood test, or current treatment with insulin or oral hypoglycemic agent. Systolic and diastolic pressure of the brachial artery was measured with a Baumanometer mercury sphygmomanometer (WA Baum Co., Inc., Copiague, NY, USA), in accordance with standards proposed by the American Heart Association, while the subjects were seated in a chair after resting quietly for over 10 minutes.

#### 2.2.2. Metabolic Parameters

Blood was collected from all subjects on the morning of the test day after an 8 h fast. Fasting blood glucose, total cholesterol, triglyceride (TG), HDL-cholesterol, and LDL-cholesterol were measured with a Hitachi Automatic Analyzer 7600 (Hitachi, Tokyo, Japan). AST, ALT, gamma-glutamyl transferase (GGT), and serum creatinine levels were measured through a blood test by using an ADVIA 1650 automatic analyzer (Bayer Diagnostics, Leverkusen, Germany).

#### 2.2.3. Plant Materials

To select the best barley cultivar of the barley (*Hordeum vulgare* L.) species for extract production, 10 types of barley cultivars were purchased from the National Institute of Crop Science (NICS; Miryang, Republic of Korea) and were germinated in a modified commercial soil bed (soil bulk density, 0.7–1.0 mg/m^3^; pH, 4.5–5.5; available phosphate, 450–650 mg/L; nitrogen, 800–1000 mg/kg; Punong Bed Soil, Punong, Korea). Daejin barley was selected as the optimal species to produce the supplement for this clinical trial. The germinated barley was grown in a growth chamber (DSGC 768, Dongseo Science, Republic of Korea) at 22-23°C with a relative humidity of 60% in a 900–1000 lux environment. Between the 13th and 20th day after germination, young barley leaves about 10–15 cm long were harvested and freeze-dried [9]. We analyzed the active constituents of barley sprout extract by using HP-5MS (5% diphenyl–95% dimethylsiloxane copolymer) capillary GC column (30 m × 0.25 *μ*m × 0.25 *μ*m film thickness; Agilent Technologies, Santa Clara, CA) and identified 20 active constituents (saponarin, lutonarin, luteolin, cynaroside, orientin, isoorientin, vitexin, isovitexin, ferulic acid, chlorogenic acid, luteolin-3-7-di-glucoside, eicosanol, heneicosanol, docosanol, tricosanol, tetracosanol, hexacosanol, heptacosanol, octacosanol, triacontanol). Among these physiologically active substances, hexacosanol, which comprises over 70% of the policosanol compounds in the extract, was the most likely constituent of barley sprout extract to improve blood lipid metabolism and prevent atherosclerotic complications [9]. For the clinical trial, freeze-dried barley sprout extract was ground into a powder and a special clinical formulation manufacturer (Silla Biotech, Pusan, Korea) used the powder to make a clinical formulation capsule (500 mg/capsule). The total policosanol content per capsule was confirmed to be about 7.5 ± 0.3 mg, which is in the scope of the 5–20 mg recommended daily dose of policosanol. To evaluate the safety of the barley sprout extract for this clinical trial, a 3-(4,5-dimethylthiazol-2-yl)-2,5-diphenyltetrazolium bromide (MTT) assay was carried out in Dulbecco's Modified Eagle Medium (Invitrogen, Carlsbad, Canada) using HepG2 cells (Korean Cell Line Bank, Seoul, Republic of Korea) and cell viability was measured as absorbance at 550 nm using a microplate reader (Molecular Devices, Sunnyvale, CA). No cytotoxicity was observed after 48–72 h of treatment, even at the maximal concentration of 1,000 *μ*g/mL; thus, this clinical trial could proceed.

#### 2.2.4. Study Design

Research subjects were classified into a test group and a control group in accordance with a double-blind, random assignment. Each subject consumed one capsule of barley sprout extract or placebo containing dextrin once daily for a total of 12 weeks. Anthropometric measurements and blood tests were performed during the 8th and 12th weeks of the intervention period. Compliance was confirmed at these visits by assessing the remaining amount of the test drug or placebo capsules. To monitor adverse reactions, research subjects were asked to make a voluntary report immediately after the occurrence of any symptom arising during the intervention period. The presence of adverse reactions was confirmed during interviews with a doctor and questionnaires throughout the study. The type, degree, result, and possible causes of adverse reactions were evaluated. Of the 66 research subjects who were enrolled for this clinical trial, a total of 15 dropped out either for personal reasons or because of an adverse reaction to the drug that occurred during the follow-up period; these 15 subjects were excluded and a total of 51 subjects included the analyses of the final results (Figure 1).

#### 2.2.5. Statistical Analysis

For all the measured values, nominal variables were reported as mean ± standard deviation, and ordinal categorical variables were indicated using a percentile (%). Values were rounded up to the nearest hundredth. A frequency analysis was used for general characteristics, while a paired sample *t*-test was used within each group to compare differences before and after the intervention. To analyze differences between the test group and the control group at the study endpoint, an independent sample *t*-test was carried out. A *p* value below 0.05 was considered statistically significant and all statistical analyses were performed with SAS PASW Statistics 18 software (SPSS, Chicago, IL, USA).

## 3. Results

### 3.1. General Characteristics of the Subjects

A total of 51 participants completed this study. The test group (*n* = 25) comprised 1 man and 24 women, while the control group (*n* = 26) comprised 2 men and 24 women. The average age of the test and the control groups was similar (42.48 ± 13.58 years and 40.54 ± 11.10 years, resp.). The physical examination revealed that the anthropometric measurements did not show statistically significant differences. The demographic characteristics were also similar between both groups (Table 1).

### 3.2. Effects on Laboratory Profile between Both Groups

**Figure 2:**
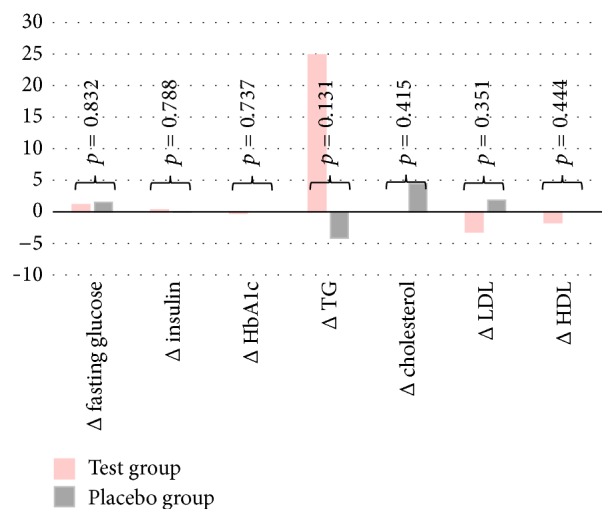
Comparisons of the differences between initial and after 12 weeks according to the groups. Delta (Δ) means differences between initial and after 12 weeks. Δ fasting glucose, mg/dL; Δ insulin, uU/dL; Δ HbA1c, %; Δ TG, mg/dL; Δ cholesterol, mg/dL; Δ LDL, mg/dL; Δ HDL mg/dL. TG, triglyceride; LDL, low density lipoprotein; HDL, high density lipoprotein. *p* values were calculated by Student's *t*-test for continuous variable.

Total cholesterol did not change after 12 weeks of taking barley sprout extract in the test group (Table 2 and Figure 2); however, LDL-cholesterol decreased by 3.2 mg/dL, from 111.16 ± 26.83 mg/dL to 107.96 ± 30.21 mg/dL. Fasting blood glucose, an index of glucose metabolism, increased by 1.2 mg/dL after taking the extract; however, glycated hemoglobin declined slightly, but not significantly, from 5.74 ± 0.36% to 5.72 ± 0.37%. Nevertheless, no statistically significant differences in any index, or in the TG, AST, ALT, and creatinine levels, were observed between both groups. Accordingly, barley sprout extract intake was confirmed not to have any meaningful effect on blood cholesterol metabolism. Based on these results, an independent sample *t*-test was executed to see whether there was any difference in lipid metabolism between both groups after the 12th week of taking the extract supplement. The differences between both groups in terms of total cholesterol and LDL-cholesterol levels after the 12th week were approximately 4.5 mg/dL and 5 mg/dL, respectively. Consequently, there was a trend for a positive effect of barley extract on lipid metabolism; however, it was not at a statistically significant level (total cholesterol, *p* = 0.415; LDL-cholesterol, *p* = 0.351; Table 3, Figure 2).

### 3.3. Treatment Tolerability

Seven of the 15 people who stopped participating in the trial complained of slight pruritus and the remaining 8 people dropped out for personal reasons. Pruritus was confirmed to be mild in all cases and improved naturally within several days, without any significant aftereffects. The pruritus did not require a prescription drug, such as an antihistamine, for mitigating the symptom; however, all the affected individuals declined to continue their participation in the clinical trial. Comparisons of patient compliance, adverse reactions, and tolerance of the extract yielded no statistically significant differences between both groups (Table 4).

## 4. Discussion

This study investigated how the intake of a test drug containing barley sprout extract for 12 weeks in healthy adults affected blood lipid levels. Accordingly, the aim of this clinical trial was to follow up on these findings in a larger group of people; however, no statistically significant improvements in blood lipid metabolism were observed in a comparative analysis of 51 subjects (*n* = 25 in the test group, *n* = 26 in the control group). However, total cholesterol and LDL-cholesterol levels in the test group were slightly lower than in the control group after 12 weeks.

Functional health foods are foods manufactured and processed by using raw materials and ingredients that improve the function of the human body. If such food is proven to have an effect on the structure and function of the human body or any positive change in nutrition control or physiological function, a clinically useful effect may be expected; thus, various studies for identifying diverse functional health foods have been conducted [2]. Barley is among a variety of foods that may have a positive effect on lipid metabolism, such as improved blood cholesterol. Barley has been a main food crop of humans since 17,000–18,000 B.C. and is currently the 4th most produced grain crop in the world. Worldwide, it has been used for food, beer, and animal feed. In Korea, where people live on grains, barley has been the second most consumed grain after rice [3]. Consumption of barley has shown a temporary decline due to the westernization of dietary habits; however, as the effect of various physiologically active substances in barley has been reilluminated recently, there has been a resurgence of interest in barley [5, 9]. Moreover, the prevalence of metabolic syndromes including obesity, hyperlipidemia, diabetes, and hypertension has shown a gradual increase. Various physiologically active substances in barley are expected to exhibit anti-inflammatory, antiobesity, and antiarteriosclerotic effects beyond just the simple improvement of blood cholesterol levels of people suffering from various chronic diseases related to hyperlipidemia [2].

Barley is known to have different types and ratios of physiologically active substances in different parts of the plant, such as seed, leaf, stalk, and awn. In accordance with the latest research, barley is confirmed to have 29 types of flavonoid and 2 types of polyphenol constituents; and the barley leaf in particular has the highest level of physiologically active substances, of which the most abundant is lutonarin [5]. Aside from lutonarin, the antioxidants saponarin, which is in the barley sprout, and superoxide dismutase (SOD) are both reported to be present in high levels [6]. Policosanols, which are lipid alcoholic substances with carbon numbers of 20–30 and *β*-glucan, which is a water-soluble dietary fiber, are abundant in barley and may contribute to its cholesterol-lowering effects [7–10]. We decided to conduct research into the leaf part of sprouting barley, so barley seeds were sown after being soaked in water for about 3 h until a sprout appeared. Then, a young leaf part that had grown to 10–15 cm long was used for research. Barley leaves from a young plant at the 1–3 leaf stage are known to contain various physiologically active substances that have 5–10 times higher nutrition content and 4–100 times greater amounts of physiological active substance than those of a full-grown mature plant, despite its small size [11, 14]. When a barley seed has germinated to a sprout for the first time while overcoming the given temperature and environment, it manufactures diverse physiologically active substances to protect the gemmule from a variety of external attacks, such as from viruses, bacteria, and fungi [15].

Therefore, the sprouting part of the plant is known to contain many various physiologically active substances compared with the full-grown mature plant. In the latest research using animal models, an extract of barley sprout reduced cholesterol levels, reduced fasting blood glucose, and exhibited antioxidant effects compared to control [8, 11–13]. Barley sprout extract was reported to lower blood cholesterol levels and to contain lutonarin, which has been shown to have antioxidant effects, hexacosanol, and *β*-glucan [5, 9, 10, 14]. A meta-analysis published in 2005 drew attention for reporting that policosanol decreased LDL-cholesterol by 24% while increasing HDL-cholesterol by 11% [16]. In a study of 40 hyperlipidemic patients, the group that took barley sprout extract for 4 weeks demonstrated a reduction in total cholesterol, LDL-cholesterol, and oxidized LDL-cholesterol [17]. In a Japanese study of 36 type 2 diabetics, the group that took barley sprout extract for 4 weeks had lower total cholesterol and LDL-cholesterol [18]. In addition, a recent trial investigating policosanol conducted in 45 adults for 4 weeks confirmed similar improvements in lipid metabolism [19]. No additional improvements in blood lipid metabolism were observed in research subjects who consumed double the dose of the same policosanol extract. Subsequently, it was reported that a higher dose is not necessary.

The mechanisms by which barley sprout extract improves blood lipid metabolism have not been identified clearly, though several potential mechanisms have been proposed. Antioxidants, such as lutomarin, saponarin, and SOD, are known to be abundant in the barley leaf [5, 6]. These antioxidants are able to preserve DNA structure from degradation by free radicals and maintain the stability of the cell wall [20]. Subsequently, because barley extracts protect the human body from free radicals, they are thought to have a positive effect on blood lipid metabolism. In particular, AMP-activated protein kinase (AMPK) is known to play a key role in energy balance, homeostasis control, and lipid metabolism [21]. Accordingly, abnormal levels of AMPK are related to the risk of cardiovascular diseases, metabolic diseases, and cancer [10]. *β*-glucan, a water-soluble dietary fiber contained in barley leaf extract, is known to impair the intestinal absorption and promote the excretion of fat, which is thought to improve blood lipid metabolism and suppress tissue lipid accumulation [7].

Our clinical trial was conducted in expectation of similar improvements in lipid metabolism to that of the various studies [16–19] reviewed above; however, unlike the preliminary clinical trial, there was no clear reduction in blood cholesterol levels, which could be due to several factors. First, some of the enrolled subjects dropped out, thus limiting our sample size to 25 in the test group and 26 in the control group. We cannot exclude the possibility of selection bias because we recruited applicants through advertising at one university medical center. Because this research was undertaken for 12 weeks in community-dwelling participants, lifestyle habits including alcohol consumption, exercise, and diet, were not controlled and may have overwhelmed the effects of barley extract on lipid metabolism. It is possible that the amount of barley sprout extract, determined through animal testing and a cytotoxicity test, may not have been enough to exhibit significant action in the human body. We cannot exclude the possibility that hexacosanol in the extracts used for this clinical trial was in a glycoside form and attached to a glucose with a low absorption rate. Furthermore, the improvements in blood lipid metabolism were minimal in one study of hyperlipidemic patients who received only policosanol treatment [22]. Therefore, it is possible that beneficial effects are minimal in people with normal lipid levels and only measurable in hyperlipidemic patients or may require combination therapy with other lipid-lowering agents. To overcome these problems and obtain meaningful results, it is necessary to conduct further research into the lipid-lowering effects of barley extract in hyperlipidemic patients. Furthermore, it may be necessary to conduct a larger clinical trial with more research subjects and various barley sprout extract dosages and frequencies to more accurately assess the effect of barley sprout extract on blood lipid metabolism. It is also necessary to confirm whether the intake of barley sprout extract supplement can enhance the effects of other lipid-lowering agents in hyperlipidemic patients undergoing treatment.

The findings of this clinical trial indicate that barley sprout extract, as a functional health food, cannot replace lipid-lowering drugs, such as statins or fenofibrates. However, the positive effects that have been reported repeatedly in diverse laboratory studies, including animal models, may warrant further large-scale follow-up studies to investigate various dosing schemes in more research subjects. Barley sprout extract may improve lipid metabolism and lower cardiovascular disease risk in people with borderline cholesterol levels, although it may not replace current drug therapies.

## Figures and Tables

**Table 1:** Baseline characteristics of all participants.

	Study group (*n* = 25)	Placebo group (*n* = 26)	*p* value
Age, years	42.48 ± 13.58	40.54 ± 11.10	0.578
Height, cm	161.56 ± 5.40	163.15 ± 5.69	0.311
Weight, kg	58.52 ± 6.16	61.19 ± 10.09	0.261
BMI, kg/m^2^	22.51 ± 2.20	22.89 ± 2.98	0.605
WC, cm	76.36 ± 8.17	77.50 ± 8.38	0.625
SBP, mmHg	114.48 ± 11.87	111.28 ± 11.50	0.331
DBP, mmHg	69.72 ± 9.94	66.58 ± 10.07	0.267
Pulse, beats/min	74.44 ± 8.79	77.12 ± 10.37	0.326
Fasting glucose, mg/dL	87.88 ± 8.64	88.12 ± 7.95	0.920
Plasma insulin, uIU/dL	6.23 ± 4.07	5.89 ± 2.43	0.718
HbA1c, %	5.74 ± 0.36	5.61 ± 0.49	0.293
Triglyceride, mg/dL	86.84 ± 42.87	81.96 ± 40.95	0.890
Total cholesterol, mg/dL	182.16 ± 31.89	183.31 ± 26.70	0.692
LDL cholesterol, mg/dL	111.16 ± 26.83	115.12 ± 27.23	0.951
HDL cholesterol, mg/dL	57.56 ± 12.86	57.77 ± 11.17	0.604
AST, U/L	16.80 ± 10.57	14.27 ± 5.09	0.201
ALT, U/L	21.28 ± 12.38	18.00 ± 3.53	0.278
BUN, mg/dL	12.08 ± 4.25	11.98 ± 4.49	0.933
Creatinine, mg/dL	0.81 ± 0.09	0.79 ± 0.10	0.344
Computed tomography			
Total fat, cm^2^	272.60 ± 84.48	277.18 ± 93.86	0.856
Visceral fat, cm^2^	77.83 ± 37.96	80.83 ± 41.56	0.789
Subcutaneous fat, cm^2^	194.76 ± 64.23	196.37 ± 68.30	0.931
Body composition analysis			
Fat, %	31.06 ± 5.96	29.62 ± 5.93	0.392
Fat, kg	18.30 ± 4.54	18.41 ± 6.19	0.942
Lean body mass, kg	21.68 ± 2.55	23.22 ± 3.65	0.088

Data are expressed as mean ± standard deviation.

*p* values were calculated with Student's *t*-test for continuous variables.

BMI, body mass index; WC, waist circumference; SBP, systolic blood pressure; DBP, diastolic blood pressure; LDL, low density lipoprotein; HDL, high density lipoprotein; AST, aspartate aminotransferase; ALT, alanine aminotransferase; BUN, blood urea nitrogen.

**Table:** (a) Test group (*n* = 25)

	Baseline	After 12 weeks	*p* value
Fasting glucose, mg/dL	87.88 ± 8.64	89.00 ± 8.38	0.416
Plasma insulin, *μ*U/dL	6.23 ± 4.07	6.52 ± 4.62	0.804
HbA1c, %	5.74 ± 0.36	5.72 ± 0.37	0.662
Triglyceride, mg/dL	86.84 ± 42.87	111.52 ± 114.27	0.190
Total cholesterol, mg/dL	182.16 ± 31.89	182.16 ± 33.19	1.000
LDL cholesterol, mg/dL	111.16 ± 26.83	107.96 ± 30.21	0.453
HDL cholesterol, mg/dL	57.56 ± 12.86	55.84 ± 12.67	0.313

**Table:** (b) Placebo group (*n* = 26)

	Baseline	After 12 weeks	*p* value
Fasting glucose, mg/dL	88.12 ± 7.95	89.61 ± 6.24	0.210
Plasma insulin, *μ*U/dL	5.89 ± 2.43	5.85 ± 1.97	0.927
HbA1c, %	5.61 ± 0.49	5.61 ± 0.42	1.000
Triglyceride, mg/dL	81.96 ± 40.95	77.81 ± 45.09	0.449
Total cholesterol, mg/dL	183.31 ± 26.70	187.77 ± 28.20	0.231
LDL cholesterol, mg/dL	115.12 ± 27.23	116.96 ± 30.05	0.588
HDL cholesterol, mg/dL	57.77 ± 11.17	57.77 ± 9.99	1.000

Data are expressed as means ± standard deviations.

*p* values were calculated with paired *t*-test for continuous variables.

LDL, low density lipoprotein; HDL, high density lipoprotein.

**Table 3:** Changes between baseline and 12-week measurements.

	Test group (*n* = 25)	Placebo group (*n* = 26)	*p* value
Fasting glucose, mg/dL	1.12 ± 6.67	1.50 ± 5.95	0.832
Plasma insulin, *μ*U/dL	0.30 ± 5.91	−0.04 ± 2.12	0.788
HbA1c, %	−0.24 ± 0.27	0 ± 0.23	0.737
Triglyceride, mg/dL	24.88 ± 92.15	−4.16 ± 27.54	0.131
Total cholesterol, mg/dL	0 ± 20.21	4.46 ± 18.54	0.415
LDL cholesterol, mg/dL	−3.20 ± 20.99	1.85 ± 17.16	0.351
HDL cholesterol, mg/dL	−1.72 ± 8.34	0 ± 7.58	0.444

Data are expressed as mean ± standard deviation.

*p* values were calculated with Student's *t*-test for continuous variables.

LDL, low density lipoprotein; HDL, high density lipoprotein.

**Table 4:** Treatment tolerability in the two groups.

	Total (*n* = 66)	Test group (*n* = 35)	Placebo group (*n* = 31)	*p* value
Stopped medication, *n* (%)	15 (22.73%)	10 (28.57%)	5 (16.13%)	0.169
Reasons for stopping, *n* (%)				
Itching sense^*∗*^	7 (10.61%)	4 (11.43%)	3 (9.68%)	0.153
Personal circumstances	8 (12.12%)	6 (17.14%)	2 (6.45%)	

^*∗*^Itching sense resolved spontaneously in several days.

*p* values were calculated with Student's *t*-test for categorical variables.
